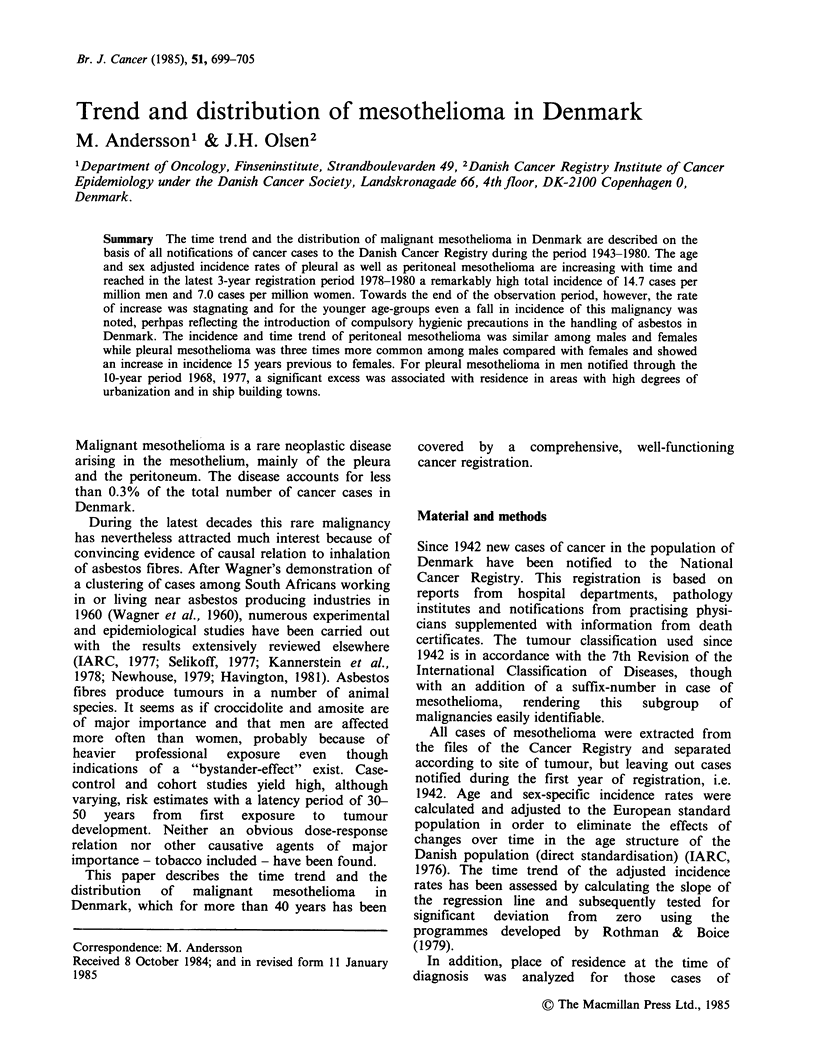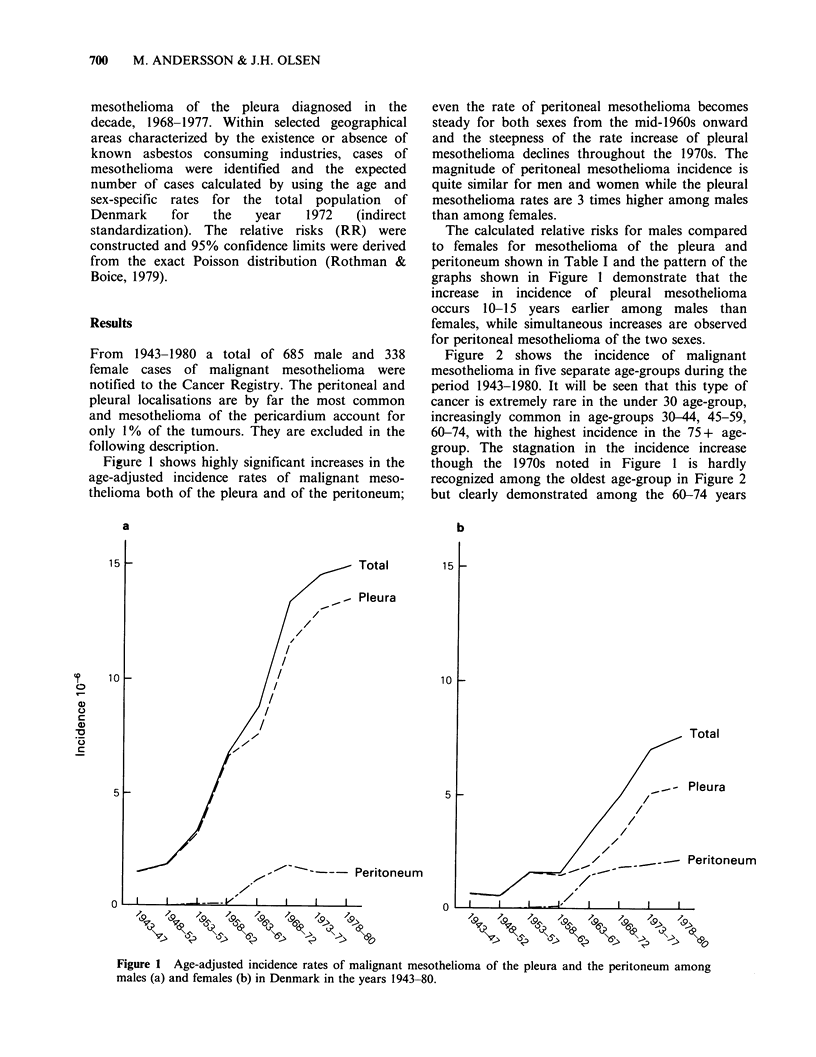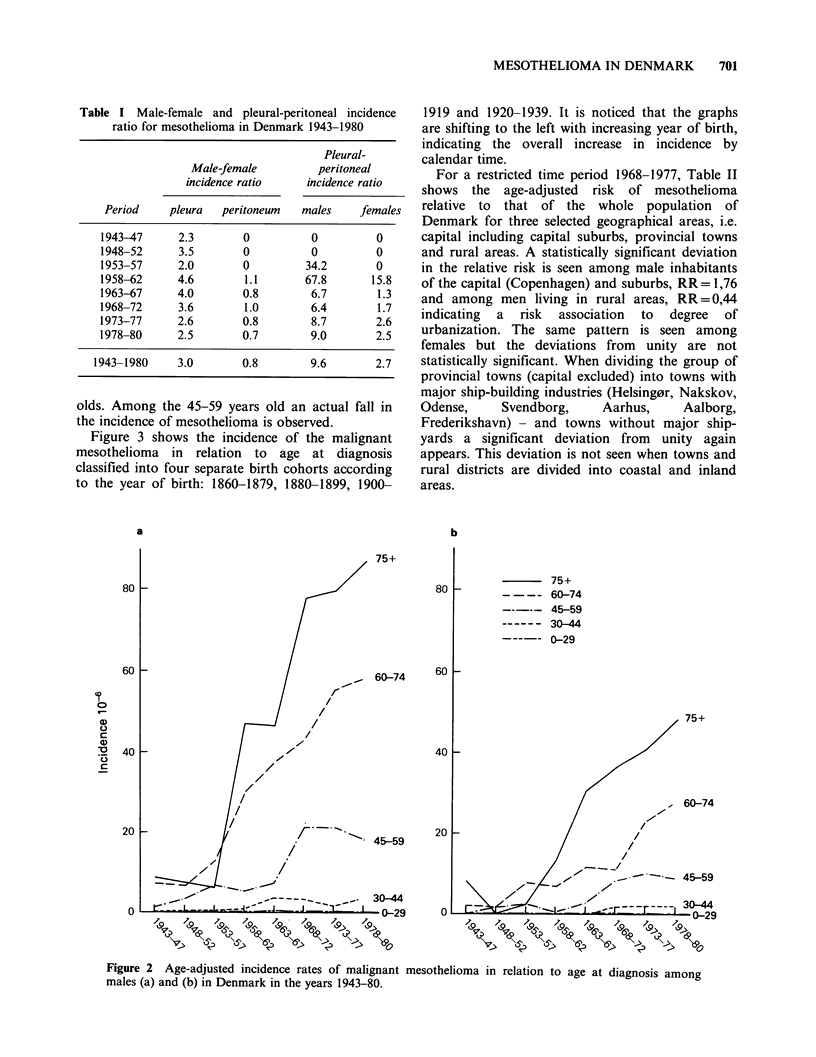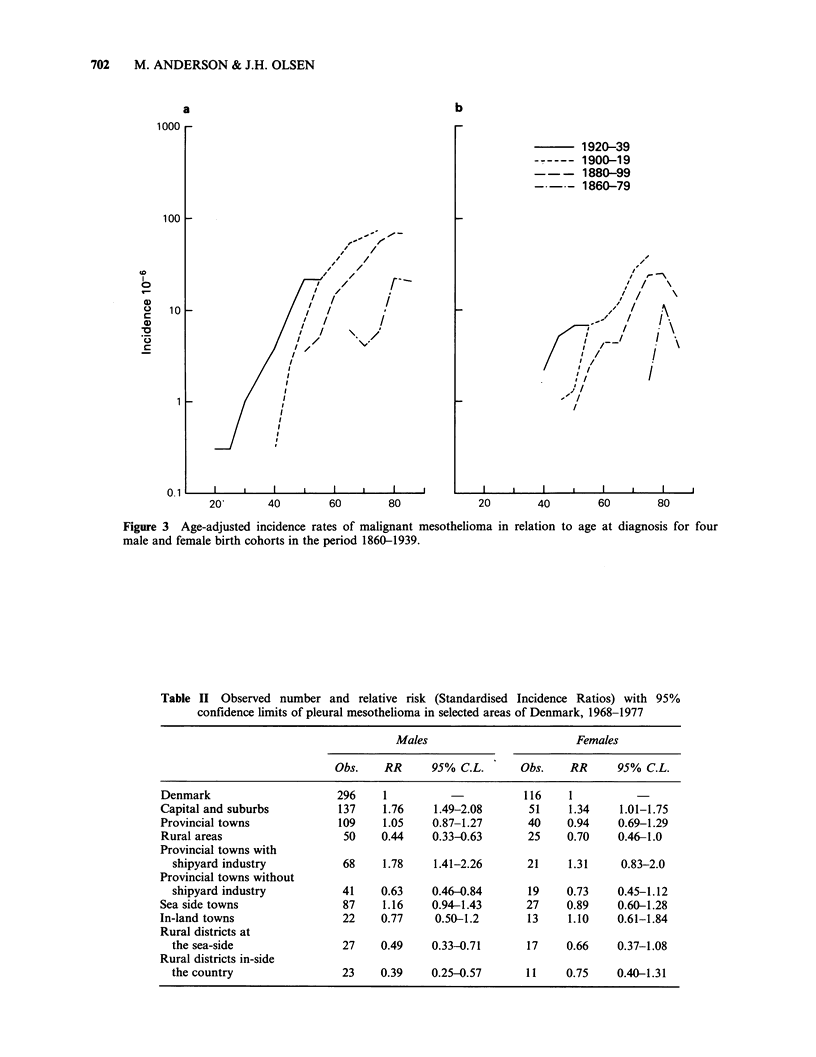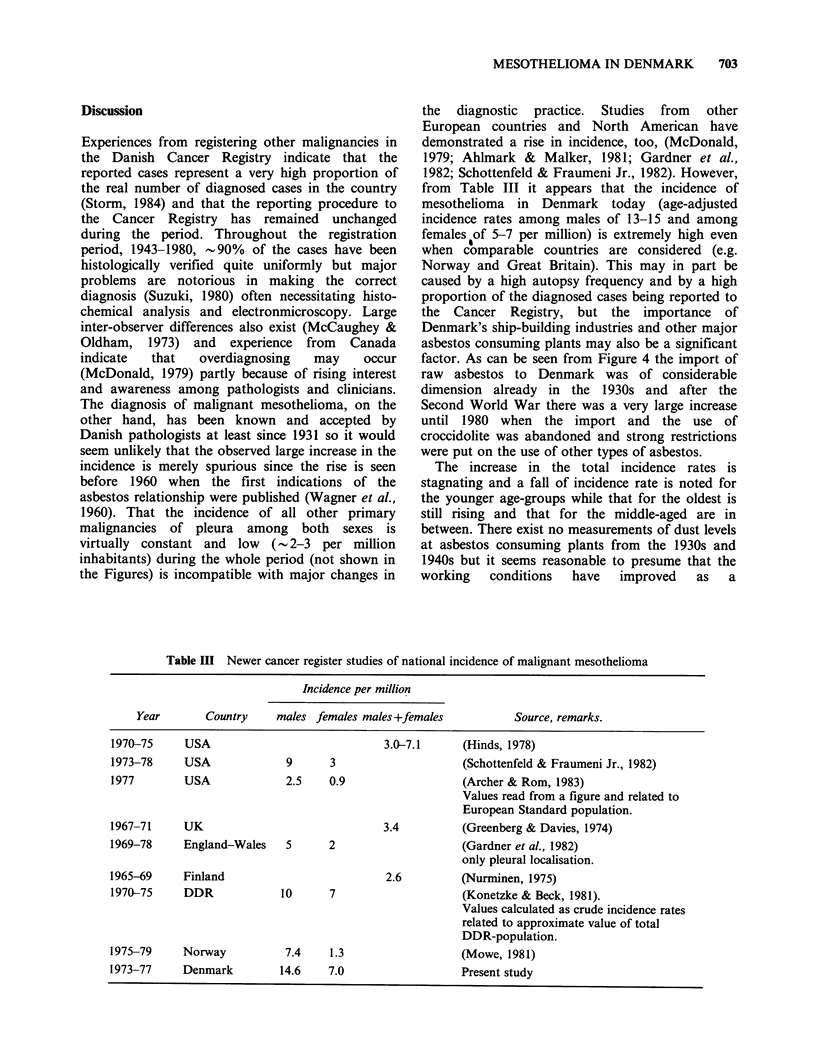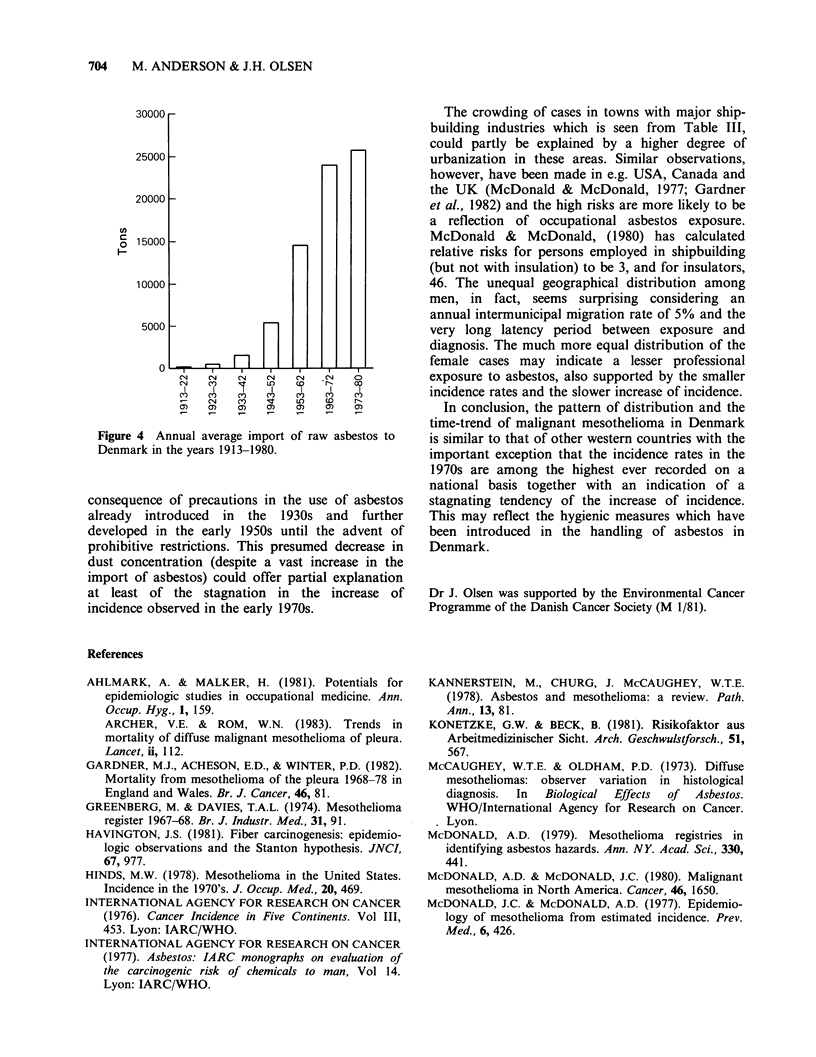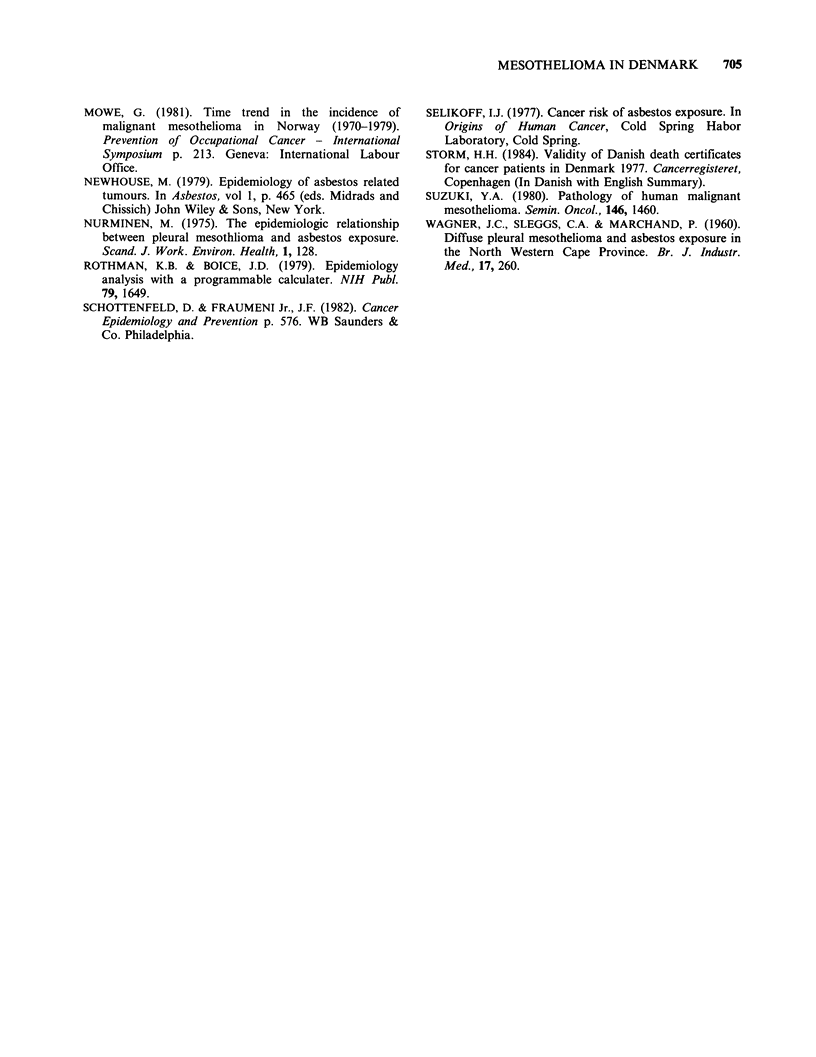# Trend and distribution of mesothelioma in Denmark.

**DOI:** 10.1038/bjc.1985.105

**Published:** 1985-05

**Authors:** M. Andersson, J. H. Olsen

## Abstract

The time trend and the distribution of malignant mesothelioma in Denmark are described on the basis of all notifications of cancer cases to the Danish Cancer Registry during the period 1943-1980. The age and sex adjusted incidence rates of pleural as well as peritoneal mesothelioma are increasing with time and reached in the latest 3-year registration period 1978-1980 a remarkably high total incidence of 14.7 cases per million men and 7.0 cases per million women. Towards the end of the observation period, however, the rate of increase was stagnating and for the younger age-groups even a fall in incidence of this malignancy was noted, perhaps reflecting the introduction of compulsory hygienic precautions in the handling of asbestos in Denmark. The incidence and time trend of peritoneal mesothelioma was similar among males and females while pleural mesothelioma was three times more common among males compared with females and showed an increase in incidence 15 years previous to females. For pleural mesothelioma in men notified through the 10-year period 1968, 1977, a significant excess was associated with residence in areas with high degrees of urbanization and in ship building towns.


*Br. J. Cancer* (1985), **51**, 699–705

### Trend and distribution of mesothelioma in Denmark

M. Andersson[1] & J.H. Olsen[2]

[1]*Department of Oncology, Finseninstitute, Strandboulevarden 49,* [2]*Danish Cancer Registry Institute of Cancer Epidemiology under the Danish Cancer Society, Landskronagade 66, 4th floor, DK-2100 Copenhagen 0, Denmark.*

**Summary** The time trend and the distribution of malignant mesothelioma in Denmark are described on the basis of all notifications of cancer cases to the Danish Cancer Registry during the period 1943–1980. The age and sex adjusted incidence rates of pleural as well as peritoneal mesothelioma are increasing with time and reached in the latest 3-year registration period 1978–1980 a remarkably high total incidence of 14.7 cases per million men and 7.0 cases per million women. Towards the end of the observation period, however, the rate of increase was stagnating and for the younger age-groups even a fall in incidence of this malignancy was noted, perhpas reflecting the introduction of compulsory hygienic precautions in the handling of asbestos in Denmark. The incidence and time trend of peritoneal mesothelioma was similar among males and females while pleural mesothelioma was three times more common among males compared with females and showed an increase in incidence 15 years previous to females. For pleural mesothelioma in men notified through the 10-year period 1968, 1977, a significant excess was associated with residence in areas with high degrees of urbanization and in ship building towns.

Malignant mesothelioma is a rare neoplastic disease arising in the mesothelium, mainly of the pleura and the peritoneum. The disease accounts for less than 0.3% of the total number of cancer cases in Denmark.

During the latest decades this rare malignancy has nevertheless attracted much interest because of convincing evidence of causal relation to inhalation of asbestos fibres. After Wagner's demonstration of a clustering of cases among South Africans working in or living near asbestos producing industries in 1960 (Wagner *et al.*, 1960), numerous experimental and epidemiological studies have been carried out with the results extensively reviewed elsewhere (IARC, 1977; Selikoff, 1977; Kannerstein *et al.*, 1978; Newhouse, 1979; Havington, 1981). Asbestos fibres produce tumours in a number of animal species. It seems as if croccidolite and amosite are of major importance and that men are affected more often than women, probably because of heavier professional exposure even though indications of a "bystander-effect" exist. Case-control and cohort studies yield high, although varying, risk estimates with a latency period of 30–50 years from first exposure to tumour development. Neither an obvious dose-response relation nor other causative agents of major importance – tobacco included – have been found.

This paper describes the time trend and the distribution of malignant mesothelioma in Denmark, which for more than 40 years has been covered by a comprehensive, well-functioning cancer registration.

Correspondence: M. Andersson
Received 8 October 1984; and in revised form 11 January 1985

### Material and methods

Since 1942 new cases of cancer in the population of Denmark have been notified to the National Cancer Registry. This registration is based on reports from hospital departments, pathology institutes and notifications from practising physicians supplemented with information from death certificates. The tumour classification used since 1942 is in accordance with the 7th Revision of the International Classification of Diseases, though with an addition of a suffix-number in case of mesothelioma, rendering this subgroup of malignancies easily identifiable.

All cases of mesothelioma were extracted from the files of the Cancer Registry and separated according to site of tumour, but leaving out cases notified during the first year of registration, i.e. 1942. Age and sex-specific incidence rates were calculated and adjusted to the European standard population in order to eliminate the effects of changes over time in the age structure of the Danish population (direct standardisation) (IARC, 1976). The time trend of the adjusted incidence rates has been assessed by calculating the slope of the regression line and subsequently tested for significant deviation from zero using the programmes developed by Rothman & Boice (1979).

In addition, place of residence at the time of diagnosis was analyzed for those cases of

© The Macmillan Press Ltd., 1985

mesothelioma of the pleura diagnosed in the decade, 1968–1977. Within selected geographical areas characterized by the existence or absence of known asbestos consuming industries, cases of mesothelioma were identified and the expected number of cases calculated by using the age and sex-specific rates for the total population of Denmark for the year 1972 (indirect standardization). The relative risks (RR) were constructed and 95% confidence limits were derived from the exact Poisson distribution (Rothman & Boice, 1979).

### Results

From 1943–1980 a total of 685 male and 338 female cases of malignant mesothelioma were notified to the Cancer Registry. The peritoneal and pleural localisations are by far the most common and mesothelioma of the pericardium account for only 1% of the tumours. They are excluded in the following description.

Figure 1 shows highly significant increases in the age-adjusted incidence rates of malignant mesothelioma both of the pleura and of the peritoneum; even the rate of peritoneal mesothelioma becomes steady for both sexes from the mid-1960s onward and the steepness of the rate increase of pleural mesothelioma declines throughout the 1970s. The magnitude of peritoneal mesothelioma incidence is quite similar for men and women while the pleural mesothelioma rates are 3 times higher among males than among females.

The calculated relative risks for males compared to females for mesothelioma of the pleura and peritoneum shown in Table I and the pattern of the graphs shown in Figure 1 demonstrate that the increase in incidence of pleural mesothelioma occurs 10–15 years earlier among males than females, while simultaneous increases are observed for peritoneal mesothelioma of the two sexes.

Figure 2 shows the incidence of malignant mesothelioma in five separate age-groups during the period 1943–1980. It will be seen that this type of cancer is extremely rare in the under 30 age-group, increasingly common in age-groups 30–44, 45–59, 60–74, with the highest incidence in the 75+ age-group. The stagnation in the incidence increase though the 1970s noted in Figure 1 is hardly recognized among the oldest age-group in Figure 2 but clearly demonstrated among the 60–74 years

**Figure 1** Age-adjusted incidence rates of malignant mesothelioma of the pleura and the peritoneum among males (a) and females (b) in Denmark in the years 1943–80.

Table I Male-female and pleural-peritoneal incidence ratio for mesothelioma in Denmark 1943–1980

| | *Male-female incidence ratio* | | *Pleural-peritoneal incidence ratio* | |
|---|---|---|---|---|
| *Period* | *pleura* | *peritoneum* | *males* | *females* |
| 1943–47 | 2.3 | 0 | 0 | 0 |
| 1948–52 | 3.5 | 0 | 0 | 0 |
| 1953–57 | 2.0 | 0 | 34.2 | 0 |
| 1958–62 | 4.6 | 1.1 | 67.8 | 15.8 |
| 1963–67 | 4.0 | 0.8 | 6.7 | 1.3 |
| 1968–72 | 3.6 | 1.0 | 6.4 | 1.7 |
| 1973–77 | 2.6 | 0.8 | 8.7 | 2.6 |
| 1978–80 | 2.5 | 0.7 | 9.0 | 2.5 |
| 1943–1980 | 3.0 | 0.8 | 9.6 | 2.7 |

olds. Among the 45–59 years old an actual fall in the incidence of mesothelioma is observed.

Figure 3 shows the incidence of the malignant mesothelioma in relation to age at diagnosis classified into four separate birth cohorts according to the year of birth: 1860–1879, 1880–1899, 1900–1919 and 1920–1939. It is noticed that the graphs are shifting to the left with increasing year of birth, indicating the overall increase in incidence by calendar time.

For a restricted time period 1968–1977, Table II shows the age-adjusted risk of mesothelioma relative to that of the whole population of Denmark for three selected geographical areas, i.e. capital including capital suburbs, provincial towns and rural areas. A statistically significant deviation in the relative risk is seen among male inhabitants of the capital (Copenhagen) and suburbs, RR = 1,76 and among men living in rural areas, RR = 0,44 indicating a risk association to degree of urbanization. The same pattern is seen among females but the deviations from unity are not statistically significant. When dividing the group of provincial towns (capital excluded) into towns with major ship-building industries (Helsingør, Nakskov, Odense, Svendborg, Aarhus, Aalborg, Frederikshavn) – and towns without major ship-yards a significant deviation from unity again appears. This deviation is not seen when towns and rural districts are divided into coastal and inland areas.

Figure 2 Age-adjusted incidence rates of malignant mesothelioma in relation to age at diagnosis among males (a) and (b) in Denmark in the years 1943–80.

**Figure 3** Age-adjusted incidence rates of malignant mesothelioma in relation to age at diagnosis for four male and female birth cohorts in the period 1860–1939.

**Table II** Observed number and relative risk (Standardised Incidence Ratios) with 95% confidence limits of pleural mesothelioma in selected areas of Denmark, 1968–1977

| | *Males* | | | *Females* | | |
|---|---|---|---|---|---|---|
| | *Obs.* | *RR* | *95% C.L.* | *Obs.* | *RR* | *95% C.L.* |
| Denmark | 296 | 1 | — | 116 | 1 | — |
| Capital and suburbs | 137 | 1.76 | 1.49–2.08 | 51 | 1.34 | 1.01–1.75 |
| Provincial towns | 109 | 1.05 | 0.87–1.27 | 40 | 0.94 | 0.69–1.29 |
| Rural areas | 50 | 0.44 | 0.33–0.63 | 25 | 0.70 | 0.46–1.0 |
| Provincial towns with shipyard industry | 68 | 1.78 | 1.41–2.26 | 21 | 1.31 | 0.83–2.0 |
| Provincial towns without shipyard industry | 41 | 0.63 | 0.46–0.84 | 19 | 0.73 | 0.45–1.12 |
| Sea side towns | 87 | 1.16 | 0.94–1.43 | 27 | 0.89 | 0.60–1.28 |
| In-land towns | 22 | 0.77 | 0.50–1.2 | 13 | 1.10 | 0.61–1.84 |
| Rural districts at the sea-side | 27 | 0.49 | 0.33–0.71 | 17 | 0.66 | 0.37–1.08 |
| Rural districts in-side the country | 23 | 0.39 | 0.25–0.57 | 11 | 0.75 | 0.40–1.31 |

### Discussion

Experiences from registering other malignancies in the Danish Cancer Registry indicate that the reported cases represent a very high proportion of the real number of diagnosed cases in the country (Storm, 1984) and that the reporting procedure to the Cancer Registry has remained unchanged during the period. Throughout the registration period, 1943–1980, ~90% of the cases have been histologically verified quite uniformly but major problems are notorious in making the correct diagnosis (Suzuki, 1980) often necessitating histochemical analysis and electronmicroscopy. Large inter-observer differences also exist (McCaughey & Oldham, 1973) and experience from Canada indicate that overdiagnosing may occur (McDonald, 1979) partly because of rising interest and awareness among pathologists and clinicians. The diagnosis of malignant mesothelioma, on the other hand, has been known and accepted by Danish pathologists at least since 1931 so it would seem unlikely that the observed large increase in the incidence is merely spurious since the rise is seen before 1960 when the first indications of the asbestos relationship were published (Wagner *et al.*, 1960). That the incidence of all other primary malignancies of pleura among both sexes is virtually constant and low (~2–3 per million inhabitants) during the whole period (not shown in the Figures) is incompatible with major changes in the diagnostic practice. Studies from other European countries and North American have demonstrated a rise in incidence, too, (McDonald, 1979; Ahlmark & Malker, 1981; Gardner *et al.*, 1982; Schottenfeld & Fraumeni Jr., 1982). However, from Table III it appears that the incidence of mesothelioma in Denmark today (age-adjusted incidence rates among males of 13–15 and among females of 5–7 per million) is extremely high even when comparable countries are considered (e.g. Norway and Great Britain). This may in part be caused by a high autopsy frequency and by a high proportion of the diagnosed cases being reported to the Cancer Registry, but the importance of Denmark's ship-building industries and other major asbestos consuming plants may also be a significant factor. As can be seen from Figure 4 the import of raw asbestos to Denmark was of considerable dimension already in the 1930s and after the Second World War there was a very large increase until 1980 when the import and the use of croccidolite was abandoned and strong restrictions were put on the use of other types of asbestos.

The increase in the total incidence rates is stagnating and a fall of incidence rate is noted for the younger age-groups while that for the oldest is still rising and that for the middle-aged are in between. There exist no measurements of dust levels at asbestos consuming plants from the 1930s and 1940s but it seems reasonable to presume that the working conditions have improved as a

**Table III** Newer cancer register studies of national incidence of malignant mesothelioma

| | | *Incidence per million* | | | |
|---|---|---|---|---|---|
| *Year* | *Country* | *males* | *females* | *males+females* | *Source, remarks.* |
| 1970–75 | USA | | | 3.0–7.1 | (Hinds, 1978) |
| 1973–78 | USA | 9 | 3 | | (Schottenfeld & Fraumeni Jr., 1982) |
| 1977 | USA | 2.5 | 0.9 | | (Archer & Rom, 1983) Values read from a figure and related to European Standard population. |
| 1967–71 | UK | | | 3.4 | (Greenberg & Davies, 1974) |
| 1969–78 | England–Wales | 5 | 2 | | (Gardner *et al.*, 1982) only pleural localisation. |
| 1965–69 | Finland | | | 2.6 | (Nurminen, 1975) |
| 1970–75 | DDR | 10 | 7 | | (Konetzke & Beck, 1981). Values calculated as crude incidence rates related to approximate value of total DDR-population. |
| 1975–79 | Norway | 7.4 | 1.3 | | (Mowe, 1981) |
| 1973–77 | Denmark | 14.6 | 7.0 | | Present study |

**Figure 4** Annual average import of raw asbestos to Denmark in the years 1913–1980.

consequence of precautions in the use of asbestos already introduced in the 1930s and further developed in the early 1950s until the advent of prohibitive restrictions. This presumed decrease in dust concentration (despite a vast increase in the import of asbestos) could offer partial explanation at least of the stagnation in the increase of incidence observed in the early 1970s.

The crowding of cases in towns with major shipbuilding industries which is seen from Table III, could partly be explained by a higher degree of urbanization in these areas. Similar observations, however, have been made in e.g. USA, Canada and the UK (McDonald & McDonald, 1977; Gardner *et al.*, 1982) and the high risks are more likely to be a reflection of occupational asbestos exposure. McDonald & McDonald, (1980) has calculated relative risks for persons employed in shipbuilding (but not with insulation) to be 3, and for insulators, 46. The unequal geographical distribution among men, in fact, seems surprising considering an annual intermunicipal migration rate of 5% and the very long latency period between exposure and diagnosis. The much more equal distribution of the female cases may indicate a lesser professional exposure to asbestos, also supported by the smaller incidence rates and the slower increase of incidence.

In conclusion, the pattern of distribution and the time-trend of malignant mesothelioma in Denmark is similar to that of other western countries with the important exception that the incidence rates in the 1970s are among the highest ever recorded on a national basis together with an indication of a stagnating tendency of the increase of incidence. This may reflect the hygienic measures which have been introduced in the handling of asbestos in Denmark.

Dr J. Olsen was supported by the Environmental Cancer Programme of the Danish Cancer Society (M 1/81).

### References

AHLMARK, A. & MALKER, H. (1981). Potentials for epidemiologic studies in occupational medicine. *Ann. Occup. Hyg.*, **1**, 159.

ARCHER, V.E. & ROM, W.N. (1983). Trends in mortality of diffuse malignant mesothelioma of pleura. *Lancet*, **ii**, 112.

GARDNER, M.J., ACHESON, E.D., & WINTER, P.D. (1982). Mortality from mesothelioma of the pleura 1968–78 in England and Wales. *Br. J. Cancer*, **46**, 81.

GREENBERG, M. & DAVIES, T.A.L. (1974). Mesothelioma register 1967–68. *Br. J. Industr. Med.*, **31**, 91.

HAVINGTON, J.S. (1981). Fiber carcinogenesis: epidemiologic observations and the Stanton hypothesis. *JNCI*, **67**, 977.

HINDS, M.W. (1978). Mesothelioma in the United States. Incidence in the 1970's. *J. Occup. Med.*, **20**, 469.

INTERNATIONAL AGENCY FOR RESEARCH ON CANCER (1976). *Cancer Incidence in Five Continents.* Vol III, 453. Lyon: IARC/WHO.

INTERNATIONAL AGENCY FOR RESEARCH ON CANCER (1977). *Asbestos: IARC monographs on evaluation of the carcinogenic risk of chemicals to man,* Vol 14. Lyon: IARC/WHO.

KANNERSTEIN, M., CHURG, J. McCAUGHEY, W.T.E. (1978). Asbestos and mesothelioma: a review. *Path. Ann.*, **13**, 81.

KONETZKE, G.W. & BECK, B. (1981). Risikofaktor aus Arbeitmedizinischer Sicht. *Arch. Geschwulstforsch.*, **51**, 567.

McCAUGHEY, W.T.E. & OLDHAM, P.D. (1973). Diffuse mesotheliomas: observer variation in histological diagnosis. In *Biological Effects of Asbestos.* WHO/International Agency for Research on Cancer. Lyon.

McDONALD, A.D. (1979). Mesothelioma registries in identifying asbestos hazards. *Ann. NY. Acad. Sci.*, **330**, 441.

McDONALD, A.D. & McDONALD, J.C. (1980). Malignant mesothelioma in North America. *Cancer*, **46**, 1650.

McDONALD, J.C. & McDONALD, A.D. (1977). Epidemiology of mesothelioma from estimated incidence. *Prev. Med.*, **6**, 426.

MOWE, G. (1981). Time trend in the incidence of malignant mesothelioma in Norway (1970–1979). *Prevention of Occupational Cancer – International Symposium* p. 213. Geneva: International Labour Office.

NEWHOUSE, M. (1979). Epidemiology of asbestos related tumours. In *Asbestos,* vol 1, p. 465 (eds. Midrads and Chissich) John Wiley & Sons, New York.

NURMINEN, M. (1975). The epidemiologic relationship between pleural mesothlioma and asbestos exposure. *Scand. J. Work. Environ. Health,* **1,** 128.

ROTHMAN, K.B. & BOICE, J.D. (1979). Epidemiology analysis with a programmable calculater. *NIH Publ.* **79,** 1649.

SCHOTTENFELD, D. & FRAUMENI Jr., J.F. (1982). *Cancer Epidemiology and Prevention* p. 576. WB Saunders & Co. Philadelphia.

SELIKOFF, I.J. (1977). Cancer risk of asbestos exposure. In *Origins of Human Cancer*, Cold Spring Habor Laboratory, Cold Spring.

STORM, H.H. (1984). Validity of Danish death certificates for cancer patients in Denmark 1977. *Cancerregisteret,* Copenhagen (In Danish with English Summary).

SUZUKI, Y.A. (1980). Pathology of human malignant mesothelioma. *Semin. Oncol.,* **146,** 1460.

WAGNER, J.C., SLEGGS, C.A. & MARCHAND, P. (1960). Diffuse pleural mesothelioma and asbestos exposure in the North Western Cape Province. *Br. J. Industr. Med.,* **17,** 260.